# New enzymatically polymerized copolymers from 4-*tert*-butylphenol and 4-ferrocenylphenol and their modification and inclusion complexes with β-cyclodextrin

**DOI:** 10.3762/bjoc.8.238

**Published:** 2012-12-04

**Authors:** Adam Mondrzyk, Beate Mondrzik, Sabrina Gingter, Helmut Ritter

**Affiliations:** 1 Heinrich-Heine-Universität Düsseldorf, Institut für Organische Chemie und Makromolekulare Chemie, Universitätsstrasse 1, 40225 Düsseldorf, Germany, Fax: (+49) 211-811-5840

**Keywords:** copolymer, ß-cyclodextrin, enzymatic polymerization, 4-ferrocenylphenol, polyphenol, 4-*tert*-butylphenol, horseradish peroxidase, HRP oxidative coupling, inclusion complexes, polymer-analogous modification

## Abstract

The enzymatically catalyzed synthesis of a copolymer of 4-*tert*-butylphenol and 4-ferrocenylphenol by horse radish peroxidase (HRP) in the presence of H_2_O_2_ in a 1,4-dioxane/water system is described. Furthermore, polymer-analogous alkylation of the free hydroxy groups and subsequent click reaction with mono-6-azido-6-desoxy-β-cyclodextrin (N_3_-β-CD) was carried out. The formation of inter- and intramolecular inclusion complexes was investigated by DLS measurement.

## Introduction

Polyphenols in general play an important role in nature, e.g., lignin and suberin. In industry, Bakelite represents the first practically successful polyphenol resin [1]. In the latter case, the phenol moieties are covalently connected by formaldehyde condensation. Alternative methods include electrochemical polymerization, metal catalysis or treatment with enzymes, which lead to phenyl–phenyl connected polyphenols [2–5]. In particular, oxidoreductase enzymes have the great advantage of nontoxicity and regioselective phenol–phenol coupling [6–8]. Several oxido-reductases and their catalytic mechanisms are well-studied; the most common are soybean peroxidase, bilirubin peroxidase, laccase and horse radish peroxidase (HRP) [8–12].

Taking the latter case, horse radish peroxidase catalyzes the oxidative polycondensation of electron-rich phenols in water/organic-solvent systems in the presence of hydrogen peroxide. In recent studies it was demonstrated that several *para*-substituted phenols, i.e., 4-*tert*-butylphenol, can be polymerized with HRP in high yield and relatively high molecular weights [13]. Also several polyphenols with further functional groups, such as nitrones, double bonds and imides, have been synthetized and investigated in our group [14–18]. There are also several natural phenols such as flavonoides, or isoflavonoides, which were also polymerized successfully [19].

4-Ferrocenylphenol (**2**) has been the subject of several studies, especially with regard to its electrochemical properties. After electrochemical oxidation of the Fe(II) ion to Fe(III) a one-electron transfer from the phenol moiety takes place, in which a proton is transferred simultaneously [20]. Furthermore, 4-ferrocenylphenol shows an antianemic activity in mongrel rabbits [21].

Metal-containing polymers have drawn a lot of interest in the past decade because of their combined chemical, electrochemical, magnetic and optic properties. Potential applications include polymeric materials for light-emitting electrodes, solar cells or field-effect transistors [22].

Attempts to polymerize **2** in the presence of enzymes have been carried out by several research groups but lead to undesired, electrochemically inactive products [23]. Nevertheless, the copolymerization of **2** and 4-*tert*-butylphenol (**3**) to obtain electrochemically active products has not been investigated until now. Hence, in the present paper we report the HRP-catalyzed synthesis of novel copolymers from **2** and **3**. Click reaction of the propargyl modified polyphenol **5** with mono-(6-azido-6-deoxy)-β-cyclodextrin (**6**) was also investigated. Based on former studies, host–guest structures were created [24–30].

## Results and Discussion

**Scheme 1:**
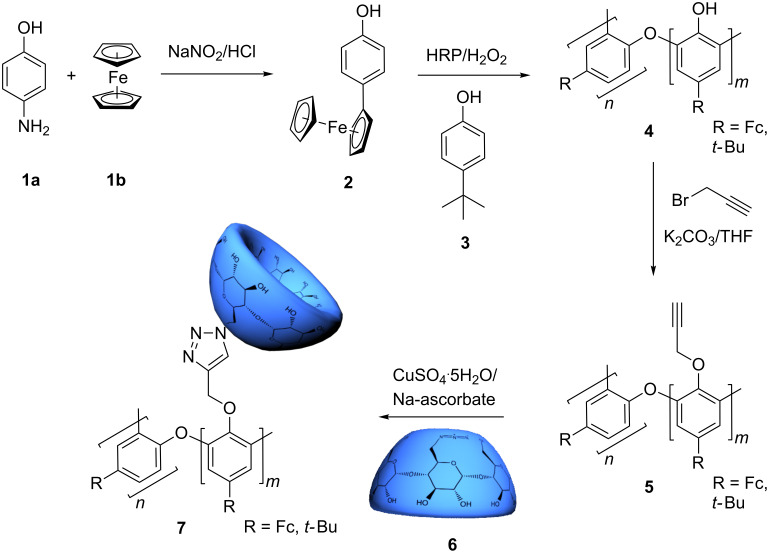
Synthetic pathway to the desired polymer **7**.

4-Ferrocenylphenol (**2**) was prepared according to the literature [31], in high purity (Scheme 1). It was then enzymatically copolymerized with 4-*tert*-butylphenol (**3**) in the presence of HRP and H_2_O_2_. The resulting copolymer **4** with a weight-average molecular weight of 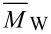 = 6000 g/mol and a dispersity of *D* = 2.1 (SEC) was received. The ^1^H NMR of the copolymer gave a broad signal in the range of 4.54–3.86 ppm (ferrocenyl group) and a broad signal between 1.79–0.06 ppm originating from the protons of the *tert*-butyl group.

As suggested in former studies, the enzyme-catalytic polymerization of phenols in general with HRP as catalyst leads to the formation of a copolymer containing phenylene and oxyphenylene moieties [32–33]. Accordingly, in the range of 1024–1174 cm^−1^ IR signals of the C–O stretching vibration of ether functionalities can be observed in the IR spectrum of copolymer **4**. Therefore, **4** is a copolymer comprising the *ortho*–*ortho*- and oxy-*ortho*-connected phenyl moieties.

The modified copolymer **5** was synthetized from copolymer **4** by alkylation with propargyl bromide under alkaline conditions. Then, the alkyne-functionalized copolymer **5** was combined successfully with mono-6-azido-6-deoxy-β-cyclodextrin (**6**) in a click-type reaction to give copolymer **7** (Scheme 1). ROESY measurements show some weak interaction of the protons of β-CD with the protons of the ferrocene (Fc) moiety and the *tert*-butyl-moiety (Supporting Information File 1).

**Figure 1:**
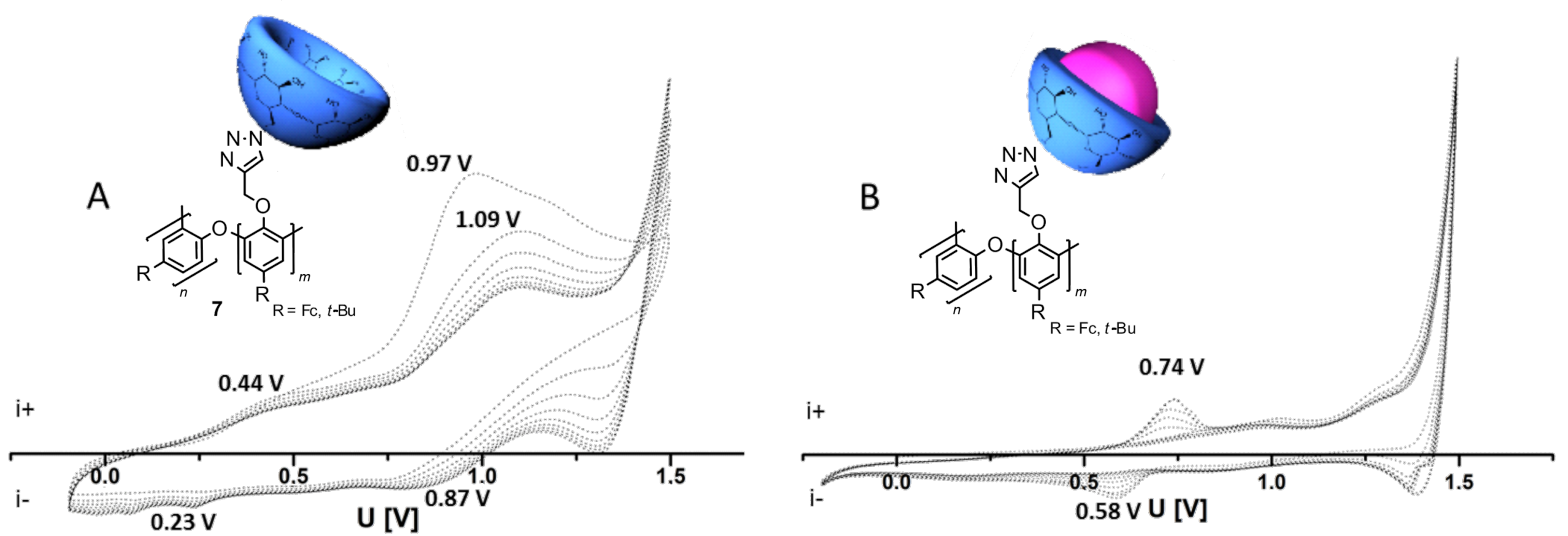
Cyclic voltammetry results of (A) clicked copolymer **7**, (B) clicked copolymer **7** with Ad-COOK (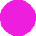).

Furthermore, the behavior of polymer **7** clicked with β-CD was studied electrochemically (Figure 1, Table 1). The cyclic voltammetry (CV) data of copolymer **7** shows an anodic peak potential at 0.97 V, which shifts to 1.09 V with each oxidation/reduction cycle due to formation of a polymer layer on the electrode (Figure 1A). The anodic peak potential at 0.44 V and both cathodic peak potentials are poorly resolved. After complexation of potassium adamantylcarboxylate (Ad-COOK) in the β-CD moieties, the CV measurements of this system show well resolved signals at 0.74 V as the anodic and 0.58 V as the cathodic peak potential. Other signals are poorly resolved (Figure 1B). In comparison, the CV measurements of **2** show anodic peak potentials of 0.66 and 0.90 V and cathodic peak potentials of 0.58 and 0.77 V. According to the literature, Fc-oxidation/reduction takes place at lower voltages than phenol oxidation/reduction [20]. The shift of peak potentials of polymerized 4-ferrocenylphenol in comparison to monomeric 4-ferrocenylphenol is +0.08 V for Fc oxidation/reduction and +0.19 V for phenol oxidation/reduction.

**Table 1:** Summary of peak potentials acquired by cyclic voltammetry (ferrocenyl oxidation or reduction/phenol oxidation or reduction).

	Anodic peak potential [V]	Cathodic peak potential [V]

**2**	0.66/0.90	0.58/0.77
**7**	0.44^a^/1.09	0.23^a^/0.87^a^
**7** + Ad-COOK	0.74/1.00^a^	0.58/0.83^a^

^a^poorly resolved signals.

In conclusion, phenol-moieties are oxidized preferably in copolymer **7**, because the ferrocenyl moieties are sterically shielded by β-CD. Accordingly, adamantylcarboxylate as a competing guest for β-CD-moieties disassembles the ferrocenyl complexes. The Fc-moieties are now readily available for oxidation/reduction on top of the electrode. This result implies successful complexation and decomplexation.

**Figure 2:**
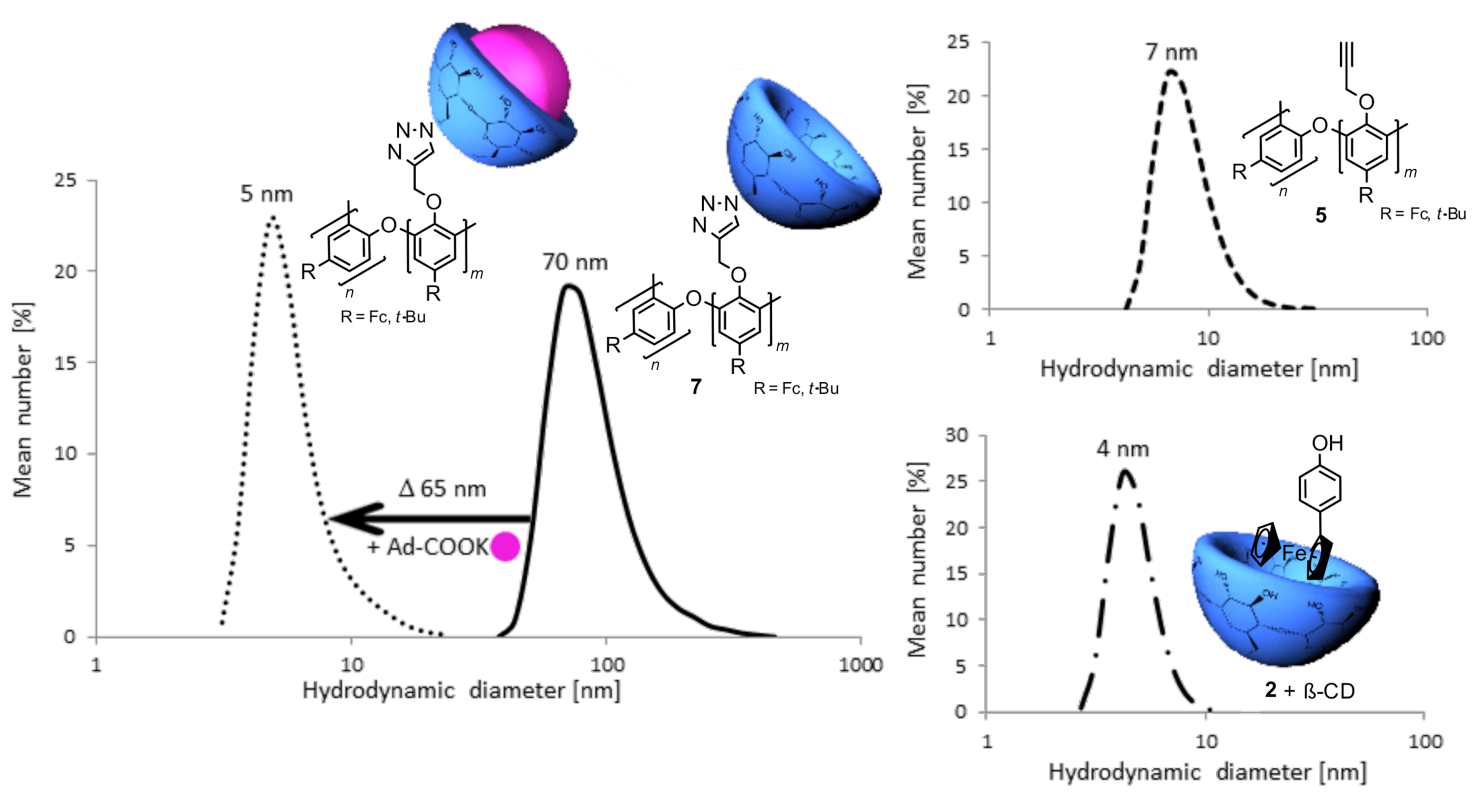
DLS measurement of compound **2** with β-cyclodextrin (— •), alkylated polyphenol **4** (- -), product **7** (—) and its complex with adamantylcarboxylate (•••).

In addition, hydrodynamic diameters also indicate strongly that supramolecular structures were formed (Figure 2). The hydrodynamic diameter of copolymer **5** (7 nm) in DMF is much smaller than the value of the cyclodextrin-modified copolymer **7** (70 nm). After breaking of supramolecular structures by addition of potassium adamantylcarboxylate as a competing guest for β-CD, the observed hydrodynamic diameter reduces to 5 nm. After measurement of compound **2** with native β-CD it is clear that self-agglomeration of the described system is a property of the modified copolymer **7**.

## Experimental

### 

#### Materials and Methods

All reagents used were commercially available (Sigma-Aldrich, Acros Organics) and were used without further purification unless stated otherwise. β-Cyclodextrin was obtained from Wacker Chemie GmbH, Burghausen, Germany, and used after drying overnight with the aid of a vacuum pump, over P_4_O_10_. Dimethylsulfoxide-*d*_6_ (99.9 atom % D) and CDCl_3_ were obtained from Deutero GmbH, Germany. 4-Ferrocenylphenol was prepared according to synthetic routes reported in the literature [31]. The inclusion complexes of potassium adamantylcarboxylate were formed by stirring a solution of the β-CD-containing polymer in DMSO with excess potassium adamantylcarboxylate overnight. The residue was then filtered off and the remaining filtrate was used for measurement.

NMR experiments (^1^H, ROESY) were performed on a Bruker Avance DRX 200 spectrometer operating at 200.13 or 500 MHz for protons with DMSO-*d*_6_ or CDCl_3_ 99.9% as solvents. The chemical shifts (δ) are given in parts per million (ppm) using the solvent peak as an internal standard.

FTIR spectra were recorded on a Nicolet 6700 FTIR spectrometer equipped with an ATR unit.

SEC measurements were performed at room temperature with THF (HPLC grade, unstabilized - Biosolv Kat.Nr. 202220602) as eluent and with a flow rate of 1 mL/min. The SEC system consists of a Gastorr BG 12 degasser (Schambeck), an Intelligent Pump AL-12 (FLOW), a S5200 sampler (Schambeck SFD) and a combination of columns (MZ Analysentechnik GmbH). The columns contain a styrene–divinylbenzene copolymer and are distributed over three main columns of porosity 10000, 1000 and 100 Å, and one column upstream of porosity 100 Å (type: Gel Sd plus). A 486 tunable absorbance detector (Waters) and a SFD RI 2000 differential-refractometer (Schambeck) were used as detectors. Calibration took place with polystyrene standards in a range of 575 to 3,114,000 g/mol. Toluene was used as the internal standard.

Dynamic light scattering (DLS) experiments were carried out with a Malvern Zetasizer Nano ZS ZEN 3600 at a temperature of 20 °C. The particle size distribution was derived from a deconvolution of the measured intensity autocorrelation function of the sample by the general purpose method (non-negative least squares) algorithm included in the DTS software. Each experiment was performed at least five times.

Microwave-assisted synthesis was performed by using a CEM Discover synthesis unit (monomode system). The temperature was measured by infrared detection with a continuous-feedback temperature control and maintained at a constant value by power modulation. Reactions were performed in closed vessels under controlled pressure.

Cyclic voltammetry (CV) measurements were performed with a computer-controlled potentiostat (SIMPOT) with a resolution in the range <100 pA. A gold electrode and an Ag/AgCl electrode were chosen as working electrode and reference electrode, respectively. The electrochemical measurements started at −0.2 V and the potential was reversed at 1.6 V. As electrolyte, a solution of 0.1 M tetra-*n*-butylammoniumhexafluorophosphate (*n*-Bu_4_N^+^PF_6_^−^) as conducting salt in DMSO was used (pH 6). Every experiment was carried out eight times.

#### Poly(4-ferrocenylphenol-co-4-*tert*-butylphenol) **4**

15 mg HRP are dissolved in 20 mL acetate buffer (pH 7); 1.39 g (5 mmol) **2** and 1.76 g (11 mmol) **3** are dissolved in 80 mL of 1,4-dioxane. The solutions are combined in a 250 mL one-neck flask and stirred vigorously. Then 1 mL of 30% hydrogenperoxide solution is added in 20 equiv portions every 15 minutes. The solution is stirred again for 12 h. The polymer precipitates to give 0.83 g (30%) yield. FTIR (diamond) 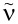: 3279, 2957, 2904, 2863, 1653, 1603, 1507, 1479, 1394, 1363, 1253, 1215, 1174, 1119, 1081, 1042, 975, 872, 828 cm^−1^; ^1^H NMR (500 MHz, CDCl_3_, 24 °C) δ 8.44–6.11 (m, 4H), 4.54–3.86 (m, 9H), 1.21 (s, 9H); SEC (THF) 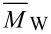 = 6000, *D* = 2.1.

#### Poly(4-ferrocenylphenol-co-4-*tert*-butylphenol)-propargyl ether **5**

0.3 g of **2** (0.7 mmol) with 0.12 g (1.0 mmol) propargyl bromide and 0.14 g (1.0 mmol) potassium carbonate are dispersed in 20 mL dry THF in a one-neck flask under an argon atmosphere. The dispersion is stirred under reflux for 24 hours. The product is precipitated in hexane and dried in vacuo to give 0.28 g (80% yield). FTIR (diamond) 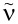: 2962, 2864, 2361, 2334, 1635, 1593, 1447, 1394, 1362, 1264, 1218, 1172, 1065 cm^−1^; ^1^H NMR (300 MHz, DMSO-*d*_6_, 25 °C) δ 8.06–6.13 (m, 4H), 4.26–4.03 (m, 11H), 3.61 (m, 2H), 1.22 (s, 9H).

#### Poly(4-ferrocenylphenol-co-4-*tert*-butylphenol-click-cyclodextrin) **7**

In a pressure vial, 0.20 g (0.41 mmol) of **5**, 0.72 g (0.62 mmol) of **6**, 0.008 g (0.03 mmol) of CuSO_4_∙5H_2_O and 0.012 g (0.06 mmol) of sodium ascorbate are dissolved in 2 mL of DMF. The reaction mixture is stirred in a microwave oven for 1 h at 90 °C (30–70 W). The product is precipitated in acetone and dried in vacuo. FTIR (diamond) 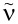: 3282, 2963, 2929, 1635, 1439, 1412, 1386, 1360, 1293, 1260, 1152, 1077, 1021 cm^−1^; ^1^H NMR (300 MHz, DMSO-*d*_6_, 25 °C) δ 8.43 (H-a), 7.47–7.13 (ArH, H-c), 5.95 (OH-2, OH-3), 4.82 (H-1), 4.55 (H-b, OH-6), 4.50–3.90 (Fc, H-d), 3.90–2.98 (H-6a,b, H-3, H-5, H-4, H-2), 1.38–0.92 (br, H-e).

## Conclusion

We described the enzymatic copolymerization of 4-ferrocenylphenol with 4-*tert*-butylphenol. The 4-ferrocenylphenol obviously tolerates the oxidative environment of H_2_O_2_. Furthermore, *ortho*–*ortho*- and oxy-*ortho*-connected phenol monomers were found. However, the free hydroxy groups were subjected to alkylation with propargyl bromide and subsequent click chemistry with N_3_-β-CD. The covalently bound cyclodextrin moiety and the covalently bound Fc or *tert*-butyl group form host/guest complexes as proven by DLS measurement. The cyclic voltammetry data shows that the central iron atom of the Fc moiety is present in the copolymer and can be oxidized and reduced, if a suitable competing guest for β-CD is present to disassemble Fc complexes.

## Supporting Information

The Supporting Information features a copy of the ROESY NMR spectrum of ferrocene- and β-cyclodextrin-containing copolymer **7**.

File 1 ROESY of copolymer **7**.
