# Supplementary material for: New enzymatically polymerized copolymers from 4-tert-butylphenol and 4-ferrocenylphenol and their modification and inclusion complexes with β-cyclodextrin
Source: Beilstein J Org Chem. 2012 Dec 4;8:2118–23. doi: 10.3762/bjoc.8.238 (PMC3520568; doi:10.3762/bjoc.8.238)

Supporting Information

for

New enzymatically polymerized copolymers from 4-*tert*-butylphenol and 4-ferrocenylphenol and their modification and inclusion complexes with β-cyclodextrin

Adam Mondrzyk, Beate Mondrzik, Sabrina Gingter, Helmut Ritter*

Address: Heinrich-Heine-Universität Düsseldorf, Institut für Organische Chemie und Makromolekulare Chemie, Universitätsstrasse 1, 40225 Düsseldorf, Germany, Fax: (+49) 211-811-5840

Email: h.ritter@uni-duesseldorf.de

*Corresponding Author

ROESY of copolymer **7**

ROESY of copolymer **7**

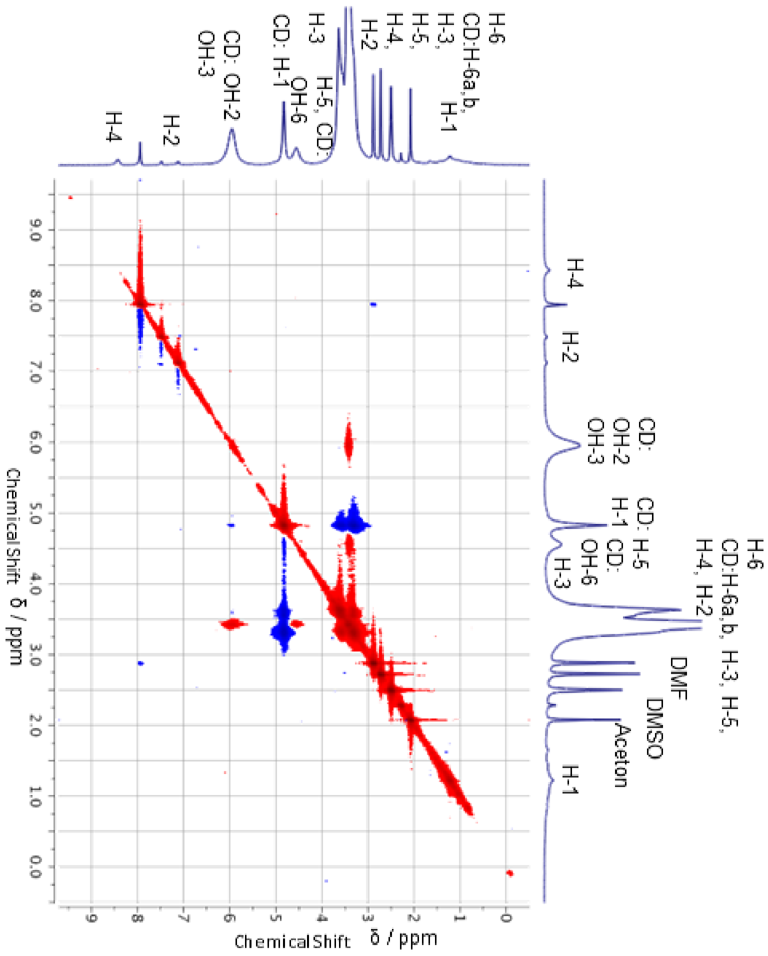

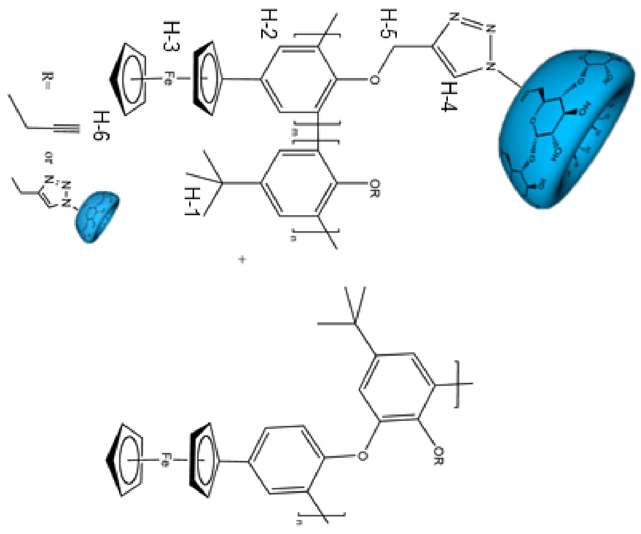

Supplement: File 1 — ROESY of copolymer 7. [file Beilstein_J_Org_Chem-08-2118-s001.pdf]
